# Association study and mutation sequencing of genes on chromosome 15q11-q13 identified *GABRG3* as a susceptibility gene for autism in Chinese Han population

**DOI:** 10.1038/s41398-018-0197-4

**Published:** 2018-08-14

**Authors:** Linyan Wang, Jun Li, Mei Shuang, Tianlan Lu, Ziqi Wang, Tian Zhang, Weihua Yue, Meixiang Jia, Yanyan Ruan, Jing Liu, Zhiliu Wu, Dai Zhang, Lifang Wang

**Affiliations:** 1 Peking University Sixth Hospital, Beijing, 100191 China; 2 Peking University Institute of Mental Health, Beijing, 100191 China; 3 Key Laboratory of Mental Health, Ministry of Health (Peking University), Beijing, 100191 China; 4 National Clinical Research Center for Mental Disorders, (Peking University Sixth Hospital), Beijing, 100191 China; 5 The Affiliated Brain Hospital of Guangzhou Medical University (Guangzhou Huiai Hospital), Guangzhou, 510370 China; 6 Peking-Tsinghua Center for Life Sciences, Peking University, Beijing, 100871 China; 7 PKU-IDG/McGovern Institute for Brain Research, Peking University, Beijing, 100871 China

## Abstract

Cytogenetic studies suggested that chromosome 15q11-q13 might be a candidate region that increases the risk of autism. Previous association studies in Caucasian populations identified the risk variants of genes in this region. However, the association of these genes with autism in Chinese Han population remains unclear. Herein, 512 autism trios were utilized for a family-based association study of 41 tag single nucleotide polymorphisms (SNPs) in this region to explore the association between protein-coding genes on chromosome 15q11-q13 and autism in Chinese Han population. Furthermore, we sequenced these autism-related genes to detect rare variants in 512 autism trios and 575 healthy controls. Our results showed that the C allele of rs7180500 in *GABRG3* was a risk variant for autism (*p* *=* 0.00057). The expression quantitative trait loci (eQTL) analysis revealed that the C allele of rs7180500 might be associated with the expression of *GABRG3* in the cerebellum (Braineac: *p* = 0.0048; GTEx: *p* = 0.0010). Moreover, the sequencing identified two rare variants rs201602655 (p.Val233Met) and rs201427468 (p.Pro365Ser) in *GABRG3* and six rare variants in *GABRB3* in autistic patients. Among these variants, rs201602655 (p.Val233Met) in *GABRG3* were observed in 9 of 512 autistic children and 2 of 575 healthy controls (Pearson *χ*^2^-test, *χ*^2^ = 5.375, *p* = 0.020). The functional prediction indicated that rs201602655 (p.Val233Met) might be deleterious. Thus, these findings demonstrated that *GABRG3* might contribute to the pathogenesis of autism in Chinese Han population.

## Introduction

Autism is a severe neurodevelopmental disorder with a typical onset before 3 years of age. The condition is primarily characterized by three abnormal symptoms: impairment in social interaction, the deficit in communication, and repetitive and restricted behaviors or interests. The prevalence of this disorder in the worldwide population is estimated at ~1%. Genetic factors were considered to play a critical role in the etiology of autism. Family and twin studies indicated that autism was highly hereditary^1,2^. Reportedly, the concordance in autism of monozygotic twins was 70%–90% as compared to ~10% in dizygotic twins^3^. However, the role of genetic factors in the pathogenesis of autism remains unclear.

Chromosome 15q11-q13 has been identified as a candidate region that increases the risk of autism^4^. This region contains several critical genes, such as GABA_A_ receptor genes cluster, *UBE3A* and *CYFIP1*, which might be correlated with the development and function of the brain^5–7^. The postmortem of autistic individuals revealed a reduced expression of *GABRB3*, *GABRA5*, and/or *GABRG3*, which was detected in several specific brain regions, such as the superior frontal cortex, parietal cortex, and cerebellum^8^. Positron emission tomography (PET) study further confirmed the reduced level of GABA_A_ receptor α5 subunit in the brains of autistic patients^9^. In addition, deficiency of GABA_A_ receptor genes cluster might be involved in autism-like behaviors. *Gabrb3*^−/−^ mice exhibited significant impairments in activities, including sociability, social novelty, and nesting, as well as tactile and heat hypersensitivity. These features were similar to the symptoms of partial autistic children^10,11^. *Gabra5*^−/−^ mice exhibited reduced social contact as well as the alterations in electroencephalograph (EEG) patterns, which were reported in autistic individuals^12,13^. Moreover, mice with overexpression of *Ube3a*, *Snprn*, or *Cyfip1* showed autism-like social deficits and repetitive self-grooming behavior^14–18^. Thus, these findings indicated that the dysfunction of genes on chromosome 15q11-q13 might play a crucial role in the pathogenesis of autism.

Previous association studies demonstrated that genes on chromosome 15q11-q13, especially GABA_A_ receptor genes cluster, might be autism susceptibility genes. Transmission disequilibrium tests (TDT) for 16 single nucleotide polymorphisms (SNPs) in GABA_A_ receptor genes cluster indicated that 2 SNPs in *GABRG3* were nominally associated with autism in Caucasians^19^. Other family-based studies detected nominally associated SNPs in *GABRB3* and *GABRA5* with autism in Europeans and Koreans^20–22^. Furthermore, a recent case-control study indicated that several SNPs and haplotypes in *GABRB3* were significantly associated with Asperger syndrome^23^, a subgroup of autism spectrum disorder (ASD). For other genes, such as *SNRPN*, *CYFIP1*, and *ATP10A*, a few SNPs or haplotypes were found to be nominally associated with autism in Europeans^20,24–26^.

In addition to common variants, rare variants might contribute to high heritability of autism. Rare and especially de novo genetic variations are known to affect liability^27–30^. Some patients with various neurodevelopmental disorders carried 15q11.2 duplication and deletion of *CYFIP1*, *NIPA2*, and *NIPA1*^31,32^. Moreover, several rare inherited variants in *GABRB3* were detected in patients affected with ASD^33^. Moreover, another study reported that a rare novel maternal transmission variant of *GABRB3* was associated with autism^34^.

In this study, we hypothesized that single nucleotide polymorphisms (SNPs) and rare mutations in the genes related to ASD and neurodevelopment in chromosome 15q11-q13 region were associated with autism. To explore the association of these genes on chromosome 15q11-q13 with autism, we performed a family-based association study between 10 protein-coding genes and autism in 512 nuclear trios of Chinese Han descent. Furthermore, we sequenced the genes on chromosome 15q11-q13 region to detect the rare variants that might contribute to the pathogenesis of autism.

## Materials and methods

### Ethics statement

This study was approved by the Ethics Committee of Peking University Sixth Hospital (China). All participants provided written informed consent to participate in this study. The informed consents of children were obtained from their legal guardians. All protocols were performed in accordance with the approved guidelines.

### Subjects

All subjects were of Chinese Han origin and recruited at Peking University Sixth Hospital China. The patients fulfilled the criteria of the Diagnostic and Statistical Manual of Mental Disorders, Fourth Edition (DSM-IV) for autistic disorder. Children with Asperger syndrome, Rett syndrome, pervasive developmental disorder not otherwise specified (PDD-NOS), fragile X syndrome, tuberous sclerosis, a previously identified chromosomal abnormality, dysmorphic features, or any other neurological condition were excluded from the study. Individuals with other familial inherited diseases or severe psychiatric disorders were also excluded. The diagnosis of autism was established by two senior psychiatrists. Autism Behavior Checklist (ABC) and Childhood Autism Rating Scale (CARS) were used to evaluate the clinical features of children^35,36^. Children with ABC score ≥53 and CARS scores ≥35 were included. A total of 512 autism nuclear trios were included in this study, of which, 449 were males and 63 were females (ratio of male: female, 7:1). The median age of the children at the time of diagnosis was 4.5 (range, 3–14) years.

We recruited age- and sex-matched healthy controls from Peking University Sixth Hospital China. A total of 575 individuals, including 480 males and 95 females, were recruited. The ratio of male to female was about 5:1. The age of healthy controls ranged from 3 to 12 years.

### SNPs selection and genotyping

**Fig. 1:**
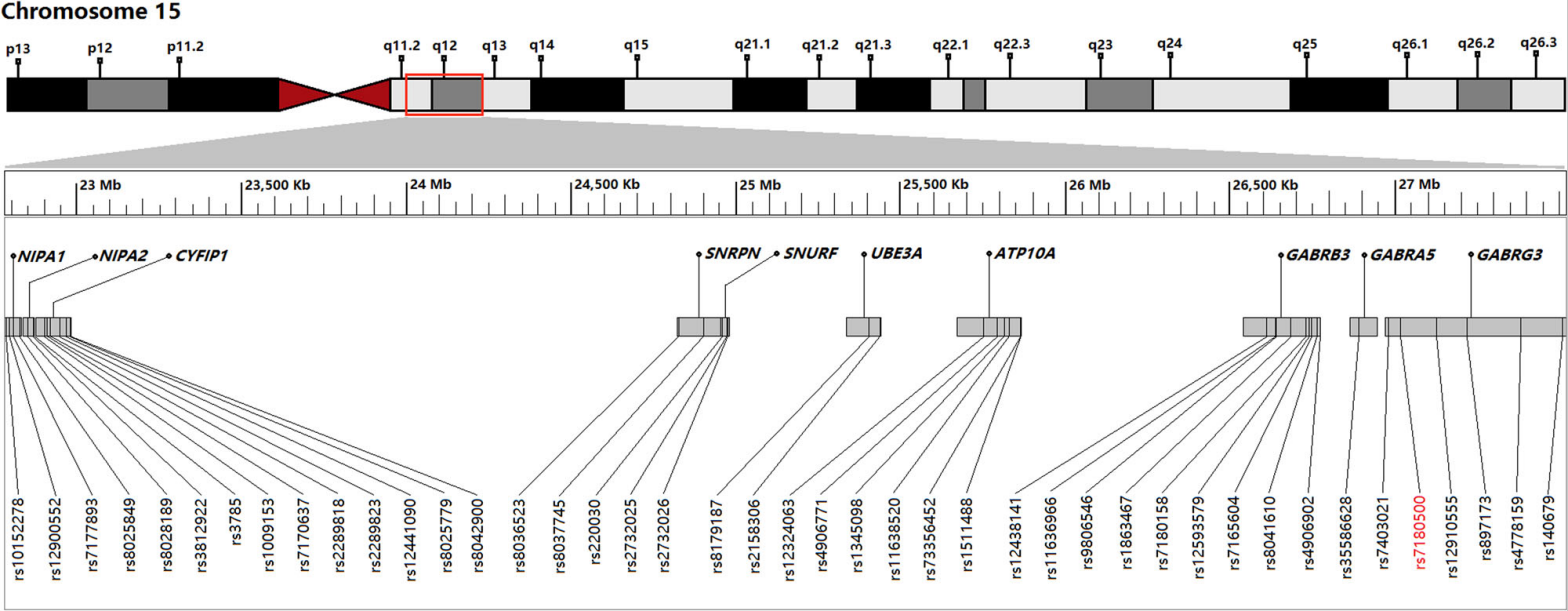
A diagram of the position of 43 selected tag SNPs located in 10 protein-coding genes in chromosome 15q11-q13. SNPs which are significantly associated with autism are noted in red color

We selected 10 protein-coding genes on 15q11-q13, which were related to ASD and brain development. The genotype data of all SNPs for Chinese Han general population in Beijing (CHB) was downloaded from the Genome Variation Server 147 (http://gvs.gs.washington.edu/GVS147) and the dbSNP in NCBI (http://www.ncbi.nlm.nih.gov/SNP/). The principles of tag SNPs selection were as follows: the minor allele frequency (MAF) value of the selected SNP should be greater than 0.05; positive-associated SNPs reported in other ethnic population and genome-wide association study (GWAS) data with autism, autism spectrum disorders type were selected; SNPs located in the functional regions of genes, such as the promoter, 5′ untranslated region (UTR), exons and 3′ UTR were preferential. After utilizing the Tagger module in Haploview version 4.2, a total of 43 tag SNPs of 10 genes were included in this study (Fig. 1). The *p*-values of Hardy–Weinberg Equilibrium (HWE) of all tag SNPs in the CHB general population were >0.05.

Genomic DNA was extracted from the blood using a Qiagen QIAamp DNA Mini Kit (Qiagen, Hilden, Germany). The genotyping of SNPs in 10 protein-coding genes was performed using the Agena Bioscience platform (Agena Bioscience, San Diego, USA), which is based on the matrix-assisted laser desorption/ionization time-of-flight (MALDI-TOF) primer extension assay^37^. Single-base primer extension with mass-modified terminators increased the plexing efficiency and flexibility of the MassARRAY system^38^. The DNA products for each SNP were prepared according to the three primary steps: DNA amplification, shrimp alkaline phosphatase (SAP) reaction and extension. All primers were designed according to the sequence of the forward strand provided by the hg19 assembly. These primers are listed in Supplementary Table S1. Subsequently, the products were dispensed into the SpectroCHIP array and the spectra of products for each SNP were acquired using the platform's mass spectrometer. Next, we randomly selected 10% samples to confirm the genotyping results using Sanger sequencing.

### Target genes capture and sequencing

A customized capture array (NimbleGen, Roche) was designed to enrich the genes on chromosome 15q11-q13 (including *NIPA1*, *NIPA2*, *CYFIP1*, *SNRPN*, *SNURF*, *UBE3A*, *ATP10A*, *GABRB3*, *GABRA5*, and *GABRG3*) according to the Build GRCh 37 assembly genome annotation of NCBI. The sequencing regions included all exons, 1 Kb upstream of the transcription start site, and 3′UTR. To avoid the nonspecific binding of genomic elements to capture arrays, RepeatMasker (http://ftp.genome.washington.edu/RM/RepeatMasker.html) was used to exclude highly repetitive elements from the probe. The method similar to the WindowMasker program was used to identify these regions. Genomic DNA was captured on a NimbleGen’ array following the manufacturer’s protocols. Briefly, the genomic DNA of participants was fragmented to a size of 200 bp by ultrasonoscope. The DNA was sheared by sonication and adaptors were ligated to the resulting fragments. Subsequently, the extracted DNA was amplified by ligation-mediated polymerase chain reaction (PCR), purified, and hybridized to the capture array at 42 °C using the manufacturer’s buffer. The resulting fragments were purified and subjected to DNA sequencing on the Illumina HiSeq2500 Analyzers platform according to the manufacturer’s protocol (Fig. S1).

### Data filtering and analyses

Illumina Pipeline (version 1.3.4) was used to generate primary data containing image analyses, error estimation and base calling. Indexed primers were used to identify different reads in the raw data. These reads were accepted when they matched to the theoretical adapter indexed sequences, as well as the theoretical primer indexed sequences with a maximum of three mismatches. Then, we excluded the unqualified sequences including low-quality reads, defined as those containing >5% Ns in the read length, reads with >30% bases with a quality score <Q20, and adapter-contaminated read pairs including the indexed sequence. The remaining sequences were termed as clean reads for analysis.

Burrows-Wheeler Aligner (BWA)^39^ was employed to align the clean reads to the human reference genome from the NCBI database (build GRCh37). Picard tool was used to convert the sequence alignment files. Variant calling was performed by the Genome Analysis Toolkit (GATK)^40^. The previously identified SNPs were determined using the NCBI dbSNP. Based on the Human Gene Mutation Database at the Institute of Medical Genetics in Cardiff (HGMD, http://www.ghmd.cf.ac.uk/) or previous literature, we identified the known disease-causing mutations. The data analysis is schematically represented in Fig. S2. In addition, SIFT (http://sift.jcvi.org), PolyPhen-2 (http://genetics.bwh.harvard.edu/pph2/), and Mutation Taster (http://www.mutationtaster.org/) were used to predict the pathogenicity of the detected mutations.

### Sanger sequencing

The mutations detected by targeted sequencing were further confirmed using Sanger sequencing. The primer design tool (https://www.ncbi.nlm.nih.gov/tools/primer-blast/) was used to design the specific primers (Table S2). PCR was performed using the 2× EasyTaq PCR SuperMix (including EasyTaq DNA polymerase, dNTP and buffer) (TransGen Biotech, Beijing, China). The optimal annealing temperature for PCR was 62 °C. The DNA sequencing was performed using a BigDye Terminator Cycle Sequencing Ready Reaction Kit with AmpliTaq DNA polymerase. The PCR fragments were separated by electrophoresis on an ABI PRISM 377–96 DNA Sequencer (Applied Biosystem, Foster City, USA).

### Statistical analysis

The power of detection of risk alleles was estimated by Quanto software version 1.2.4 (http://biostats.usc.edu/software). For the identified significant risk alleles, the relative risk was set to values calculated from the previously described formula for family-based samples^41^. *χ*^2^-test was used to analyze the deviations from the HWE for genotype frequency distributions. Single marker association tests were performed using the family-based association test (FBAT) program version 2.0.3 (http://www.biostat.harvard.edu/fbat/default.html). This program implemented a generalized statistical score to perform various transmission disequilibrium tests (TDTs). Additive and recessive inheritance models were examined utilizing these tests. The genotypes of families with Mendelian errors were detected and reset to zero by FBAT. Furthermore, Bonferroni correction was performed to reduce the rate of type I errors. The significance level was set at *p* < *α*/*n* (*α* = 0.05). All *p*-values calculated by the FBAT were two-sided. Haploview version 4.2 (http://www.broad.mit.edu/mpg/haploview/) was used to calculate the ratio of transmission to untransmission (T:U) for alleles of each selected SNP.

The frequencies of rare variants were compared between the patients and controls using Pearson *χ*^2^-test when the calculated minimum expected count was >5. While Continuity Correction test were performed when the calculated minimum expected count was >1 and <5. The significance was set at *p* < 0.05 (two-sided).

### Expression quantitative trait loci (eQTL) analysis of significantly associated SNPs and expression pattern in human brain

Two online databases were used to analyze the eQTL effects of the risk alleles of the associated SNPs. The Brain eQTL Almanac (Braineac) (http://www.braineac.org/)^42^ and Genotype-Tissue Expression (GTEx) database (http://www.gtexportal.org/)^43^ provided the eQTL data for ten primary brain regions with significantly associated SNPs, respectively. These online databases aided in exploring whether one or more risk SNPs were operating as eQTL in brain regions. Moreover, the GTEx database provided the data for genes in 53 human tissues or cells. The Human Brain Transcriptome (HBT) databases (http://hbatlas.org/pages/hbtd) provided a dynamic expression of significantly associated genes during the development and adulthood in different regions of the brain.

### In silico analysis of SNPs and mutations

We explored the annotations of SNPs and detected the mutations using HaploReg v4.1 (http://compbio.mit.edu/HaploReg) which can identify the state of chromatin, conservation, and regulatory motif alterations of risk variants and SNPs^44^. MiRWalk 2.0 (http://zmf.umm.uni-heidelberg.de/apps/zmf/mirwalk2/) and TargetScan (http://www.targetscan.org/vert_71/) were used to predict whether the detected SNPs or variants were the target regions of miRNAs. rVarBase (http://rv.psych.ac.cn/index.do)^45^ and JASPAR (http://jaspar.genereg.net/) were used to explore whether the associated SNPs and mutations were regulatory SNPs and transcription factors (TFs). Furthermore, Promoter 2.0 Prediction Server (http://www.cbs.dtu.dk/services/Promoter/) and Eukaryotic Promoter database (http://epd.vital-it.ch/) were used to identify whether the variants were located in the promoter regions.

## Results

### Quality control

Genotypes of all SNPs were clustered clearly using the Agena Bioscience platform. The call rate of each genotype was ensured to be >0.95. Among the 43 selected tag SNPs, 2 SNPs (rs7170637 and rs11636966), which are common risk variants in Europeans with ASD, were identified as rare variants in our samples (MAF < 0.05). Thus, 41 tag SNPs were qualified for analysis. The allele frequencies of these 41 tag SNPs were displayed in Table 1. Among the 512 autism trios, the power to detect the potential risk alleles for the 41 selected SNPs ranged from 0.6 to 0.99. None of the genotype distributions of these tag SNPs in unaffected parents deviated from HWE (Table S2). The genotyping concordance rate for Agena Bioscience platform and direct Sanger sequencing was >99%.

**Table 1:** Results of association analyses between 41 tag SNPs in chromosome 15q11-q13 in 512 trios by FBAT under an additive model

Gene symbol	Marker	Chromosome	Allele	Afreq	Fam	T : U^a^	S-E (S)	Var (S)	*Z*	*P* ^b^
*NIPA1*	rs10152278	15:22786139	G	0.571	382	259 : 239	10.00	124.50	0.896	0.370
			A	0.429	382	239 : 259	−10.00	124.50	−0.896	0.370
	rs12900552	15:22795211	G	0.419	372	263 : 239	14.00	126.50	1.245	0.210
			A	0.581	372	239 : 263	−14.00	126.50	−1.245	0.210
	rs7177893	15:22807275	G	0.887	170	105 : 85	10.00	47.50	1.451	0.150
			T	0.113	170	85 : 105	−10.00	47.50	−1.451	0.150
	rs8025849	15:22825211	A	0.820	256	155 : 139	7.00	74.00	0.814	0.416
			G	0.180	256	139 : 155	−7.00	74.00	−0.814	0.416
*NIPA2*	rs8028189	15:22849067	C	0.273	318	220 : 196	12.00	104.00	1.177	0.203
			G	0.727	318	196 : 220	−12.00	104.00	−1.177	0.203
	rs3812922	15:22866361	C	0.780	289	198 : 152	22.50	87.75	2.402	0.016
			A	0.220	289	152 : 198	−22.50	87.75	−2.402	0.016
	rs3785	15:22867866	G	0.628	261	190 : 170	10.00	90.00	1.054	0.292
			A	0.372	261	170 : 190	−10.00	90.00	−1.054	0.292
*CYFIP1*	rs1009153	15:22896157	G	0.456	373	267 : 229	19.50	124.75	1.746	0.081
			A	0.544	373	229 : 267	−19.50	124.75	−1.746	0.081
	rs2289818	15:22912200	G	0.588	353	254 : 211	20.50	116.75	1.897	0.058
			C	0.412	353	211 : 254	−20.50	116.75	−1.897	0.058
	rs2289823	15:22945116	C	0.907	113	67 : 55	6.50	30.75	1.172	0.241
			T	0.093	113	55 : 67	−6.50	30.75	−1.172	0.241
	rs12441090	15:22967498	G	0.720	318	205 : 191	6.50	99.25	0.625	0.514
			A	0.280	318	191 : 205	−6.50	99.25	−0.625	0.514
	rs8025779	15:22979151	C	0.650	347	238 : 220	8.50	114.75	0.793	0.427
			G	0.350	347	220 : 238	−8.50	114.75	−0.793	0.427
	rs8042900	15:22980985	G	0.169	245	145 : 142	1.50	71.75	0.177	0.859
			A	0.831	245	142 : 145	−1.50	71.75	−0.177	0.859
*SNRPN*	rs8036523	15:24824408	G	0.227	300	179 : 177	0.50	89.25	0.053	0.958
			T	0.773	300	177 : 179	−0.50	89.25	−0.053	0.958
	rs8037745	15:24902158	A	0.847	226	155 : 109	23.00	66.00	2.831	0.0046
			G	0.153	226	109 : 155	−23.00	66.00	−2.831	0.0046
*SNURF*	rs220030	15:24954621	T	0.487	376	254 : 236	8.50	122.75	0.767	0.443
			C	0.513	376	236 : 254	−8.50	122.75	−0.767	0.443
	rs2732025	15:24966663	G	0.485	390	264 : 246	8.50	127.75	0.752	0.452
			T	0.514	390	246 : 264	−8.50	127.75	−0.752	0.452
	rs2732026	15:24971341	A	0.536	369	245 : 235	5.00	120.00	0.456	0.648
			C	0.464	369	235 : 245	−5.00	120.00	−0.456	0.648
*UBE3A*	rs8179187	15:25407179	T	0.635	363	242 : 237	2.00	120.00	0.183	0.855
			G	0.365	363	237 : 242	−2.00	120.00	−0.183	0.855
	rs2158306	15:25435414	T	0.366	338	232 : 227	2.50	114.75	0.233	0.815
			C	0.634	338	227 : 232	−2.50	114.75	−0.233	0.815
*ATP10A*	rs12324063	15:25743809	G	0.601	337	240 : 207	16.00	112.00	1.512	0.131
			A	0.399	337	207 : 240	−16.00	112.00	−1.512	0.131
	rs4906771	15:25787248	C	0.487	363	270 : 212	28.00	121.50	2.540	0.011
			T	0.513	363	212 : 270	−28.00	121.50	−2.540	0.011
	rs1345098	15:25812475	T	0.796	282	181 : 141	18.50	81.25	2.052	0.040
			G	0.204	282	141 : 181	−18.50	81.25	−2.052	0.040
	rs11638520	15:25828376	T	0.532	361	250 : 223	12.50	118.75	1.147	0.251
			G	0.468	361	223 : 250	−12.50	118.75	−1.147	0.251
	rs73356452	15:25865066	C	0.883	179	102 : 100	0.00	51.00	0.000	1.000
			T	0.117	179	100 : 102	0.00	51.00	0.000	1.000
	rs1511488	15:25866774	G	0.927	125	73 : 59	7.00	33.00	1.219	0.223
			C	0.073	125	59 : 73	−7.00	33.00	−1.219	0.223
*GABRB3*	rs12438141	15:26625455	C	0.922	131	86 : 60	13.00	36.50	2.152	0.031
			T	0.078	131	60 : 86	−13.00	36.50	−2.152	0.031
	rs9806546	15:26648239	A	0.833	242	150 : 133	8.00	71.00	0.949	0.343
			G	0.167	242	133 : 150	−8.00	71.00	−0.949	0.343
	rs1863467	15:26688587	C	0.574	346	253 : 202	24.50	114.25	2.292	0.022
			T	0.426	346	202 : 253	−24.50	114.25	−2.292	0.022
	rs7180158	15:26733091	A	0.359	341	224 : 209	7.50	108.25	0.721	0.471
			G	0.641	341	209 : 224	−7.50	108.25	−0.721	0.471
	rs12593579	15:26742985	A	0.689	350	224 : 215	5.00	110.00	0.477	0.634
			C	0.311	350	215 : 224	−5.00	110.00	−0.477	0.634
	rs7165604	15:26749309	T	0.684	286	192 : 186	3.00	94.50	0.309	0.758
			C	0.316	286	186 : 192	−3.00	94.50	−0.309	0.758
	rs8041610	15:26763117	C	0.414	378	253 : 246	3.00	125.50	0.268	0.789
			A	0.586	378	246 : 253	−3.00	125.50	−0.268	0.789
	rs4906902	15:26774621	A	0.667	342	263 : 197	33.00	115.00	3.077	0.002
			G	0.333	342	197 : 263	−33.00	115.00	−3.077	0.002
*GABRA5*	rs35586628	15:26886993	T	0.595	366	254 : 226	14.00	120.00	1.278	0.201
			C	0.405	366	226 : 254	−14.00	120.00	−1.278	0.201
*GABRG3*	rs7403021	15:26970515	C	0.815	252	158 : 147	5.50	76.25	0.63	0.529
			T	0.185	252	147 : 158	−5.50	76.25	−0.63	0.529
	rs7180500	15:27008032	C	0.904	159	112 : 66	23.00	44.50	3.488	**0.00057**
			A	0.096	159	66 : 112	−23.00	44.50	−3.488	**0.00057**
	rs12910555	15:27122424	A	0.872	195	120 : 102	8.50	55.75	1.138	0.255
			G	0.128	195	102 : 120	−8.50	55.75	−1.138	0.255
	rs897173	15:27224754	A	0.581	371	263 : 229	17.00	123.50	1.530	0.126
			G	0.419	371	229 : 263	−17.00	123.50	−1.530	0.126
	rs4778159	15:27396841	A	0.684	322	217 : 177	19.50	98.75	1.962	0.050
			T	0.316	322	177 : 217	−19.50	98.75	−1.962	0.050
	rs140679	15:27527530	T	0.687	322	214 : 199	6.50	103.75	0.638	0.523
			C	0.313	322	199 : 214	−6.50	103.75	−0.638	0.523

*Afreq* allele frequency, *Fam* number of informative families, *S* test statistics for the observed number of transmitted alleles, *E(S)* expected value of S under the null hypothesis (i.e., no linkage and no association)

^a^The ratio of transmisson to untransmisson (T∶U) for each selected SNP was calculated by the Haploveiw version 4.2

^b^*P*-value with bold character means the statistical significance persists after the Bonferroni correction

### SNP association analyses

Under the additive model, the single SNP association tests demonstrated that the C allele of rs7180500 in *GABRG3* was preferentially transmitted from unaffected parents to affected offspring (rs7180500: C>A, *Z* = 3.488, *p* = 0.00057) (Table 1). The significance of this result persisted even after the Bonferroni correction (*p* = *α*/*n* = 0.05/41 = 0.0012). Under the recessive model, the C allele of rs7180500 in *GABRG3* also displayed a nominal association with autism (rs7180500: C>A, *Z* = 2.798, *p* = 0.0052). The G allele of rs4906902 in *GABRB3* was a risk variant, and the C allele of rs4906771 in *ATP10A* was a protective variant after Bonferroni correction (rs4906902: A>G, *Z* = 3.441, *p* = 0.00065; rs4906771: T>C, *Z* = −3.395, *p* = 0.00069) (Table S4). Moreover, 7 SNPs (rs3812922 in *NIPA2*, rs8037745 in *SNRPN*, rs4906771 and rs1345098 in *ATP10A*, rs12438141, rs1863467, and rs4906902 in *GABRB3*) displayed nominal association with autism under the additive model in our samples (*p* < 0.05) (Table 1). Nine SNPs (rs8025849 in *NIPA1*, rs3812922 in *NIPA2*, rs1009153 and rs2289818 in *CYFIP1*, rs8037745 in *SNRPN*, rs12438141 and rs1863467 in *GABRB3*, rs7403021 and rs7180500 in *GABRG3*) were nominally associated with autism under the recessive model (*p* < 0.05) (Table S4).

### Discovery and validation of rare variants

**Fig. 2:**
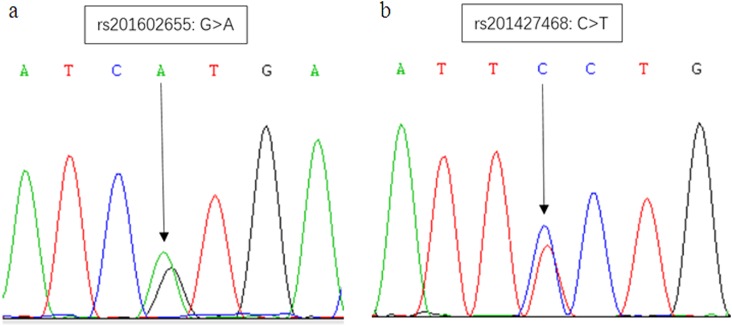
Two rare mutations in *GABRG3* detected in autistic patients validated by Sanger sequencing. The position and alleles of each SNP are indicated with an arrow

In the discovery phase, we performed targeted sequencing for the genes (including exonic coding regions and transcriptional regulation regions) on chromosome 15q11-q13 in 96 patients affected with autism. The probe coverage reached 98.1%. The average ratio of the target capture was >80% (200× depth). We detected two rare variants rs201602655 (p.Val233Met) and rs201427468 (p.Pro365Ser) in *GABRG3* in 2 autistic children and six rare single nucleotide variants (c.−693A>T, c.*417C>T, c.*704A>T, c.*1730G>A, c.*2583C>T, c.*3536T>C) in *GABRB3* in 6 of 96 patients affected with autism. These variants (all heterozygote) were validated by Sanger sequencing (Fig. 2 and Fig. S3).

Then, we expanded the sample size to 512 patients with autism and their parents in the validation phase. As for rs201602655 (p.Val233Met) in *GABRG3*, one autistic child (1/512) presented this de novo heterozygous mutation and 8 children affected with autism (8/512) were inherited from their parents (four were maternal and the others were paternal, all heterozygotes). Furthermore, we sequenced this mutation (rs201602655, p.Val233Met) in 575 age-matched healthy controls and found that 2 controls carried this heterozygous variant. The frequency of rs201602655 (p.Val233Met) in *GABRG3* in autistic children was significantly higher than that in healthy controls (9/512 vs. 2/575, Pearson *χ*^2^-test, *χ*^2^ = 5.375, *p* *=* 0.020) (Table 2).

**Table 2:** Rare variants detected in *GABRG3* in 512 autistic children

ID	Position	Nucleotide change	Amino acid change	Genotype	Variants origin			Afreq^a^	MAF^b^ (EAS/EUR/AFR/AMR/SAS)	MAF in ExAc ^c^	Prediction		
					De novo	maternal	paternal				SIFT	PolyPhen	Mutation Taster
rs201602655	15:27725918	c.697G>A	Val233Met	AG	1	4	4	0.018	0.0069/0/0.0015/0/0.0005	0.0005	Deleterious	Possibly damaging	Disease causing
rs201427468	15:27773109	c.381C>T	Pro365Ser	CT	1	0	2	0.006	0.0030/0/0/0/0.0010	0.0003	Tolerated	benign	Disease causing

*Afreq* allele frequency, *MAF* minor allele frequency

^a^Minor allele frequency in this study

^b^The MAF in East Asian (EAS), European (EUR), African (AFR), American (AMR), and South Asian (SAS)

^c^ExAc aggregated populations

Another rare variant rs201427468 (p.Pro365Ser) in *GABRG3* was detected in 3 autistic children (3/512) (one was de novo, and the other two were inherited from fathers, all heterozygotes). One healthy control also carried this mutation (1/575, heterozygote). The frequency of heterozygote of rs201427468 (p.Pro365Ser) between patients and controls demonstrated no significant difference (3/512 vs. 1/575, continuity correction *χ*^2^ = 0.382, *p* *=* 0.537) (Table 2).

All of the six rare single nucleotide variants (c.−693A>T, c.*417C>T, c.*704A>T, c.*1730G>A, c.*2583C>T, c.*3536T>C) in *GABRB3* detected in the discovery phase were inherited. When the sample size was expanded to 512 autistic children, each variant was detected only in one autistic child, respectively (Fig. S3 and Table S5).

### eQTL effects of the significantly associated SNPs and expression profile of *GABRG3* in human brain

**Fig. 3:**
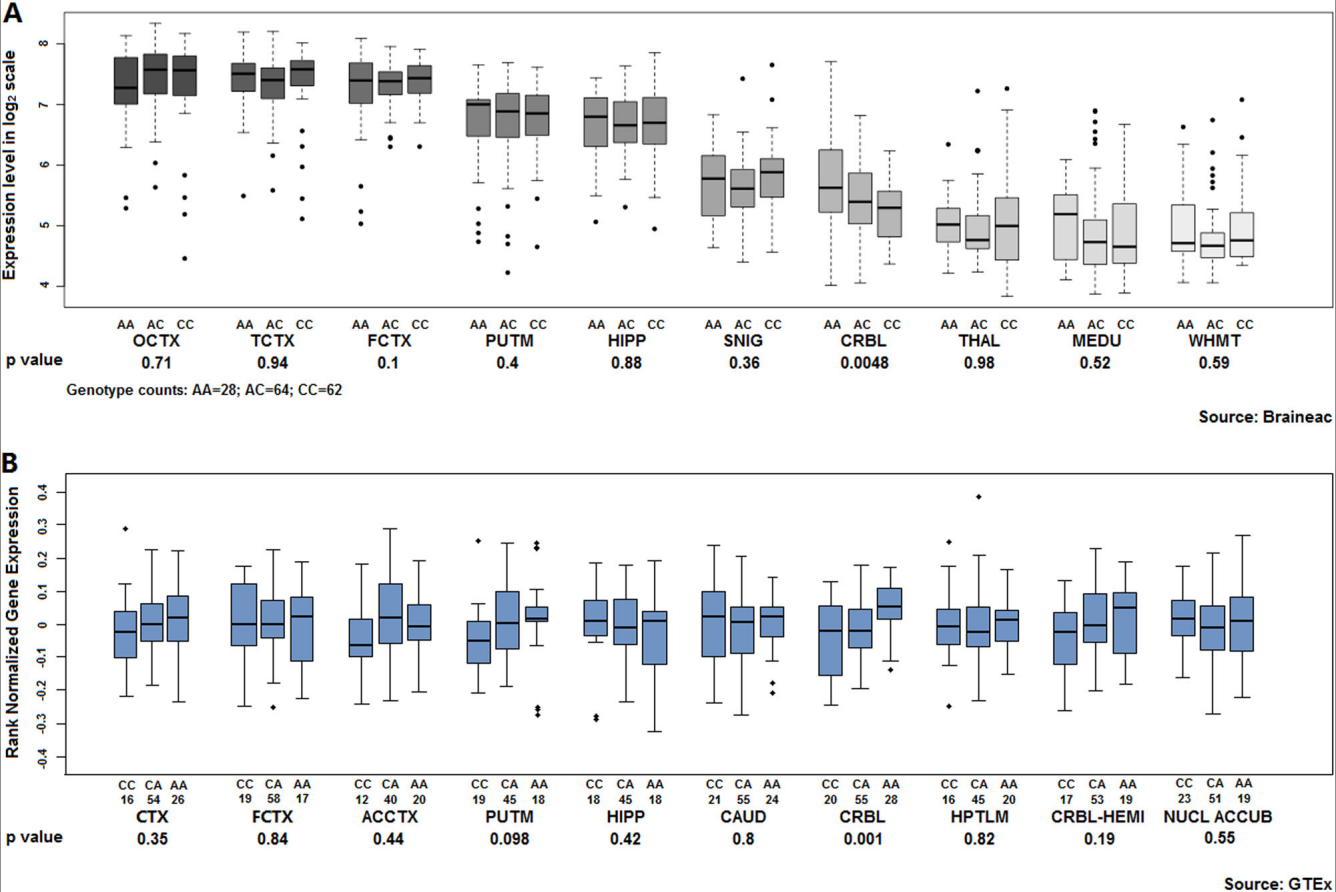
eQTL effects of rs7180500 in *GABRG3* on 10 primary brain regions from the Braineac and genotype-tissue expression (GTEx) databases. **a** WHMT white matter; MEDU medulla; PUTM putamen; THAL thalamus; SNIG substantia nigra; HIPP hippocampus; FCTX frontal cortex; TCTX temporal cortex; OCTX occipital cortex; CRBL cerebellum. **b** CTX cortex; FCTX frontal cortex; ACCTX anterior cingulate cortex; PUTM putamen; HIPP hippocampus; CAUD caudate; CRBL cerebellum; HPTML hypothalamus; CRBL-HEMI cerebellar hemisphere; NUCL ACCUB nucleus accumbens

Two online eQTL databases revealed that the C allele of rs7180500 might be associated with the expression level of *GABRG3* in the cerebellum (Braineac: *p* = 0.0048; GTEx: *p* *=* 0.0010) (Fig. 3). The data from Braineac showed that rs4906771 in *ATP10A* might exert potential eQTL effects on the frontal cortex. However, a similar result was not obtained using the GTEx databases (Braineac: *p* = 0.022; GTEx: *p* = 0.12) (Fig. S4 and Table S5).

Furthermore, the RNA-seq data from GTEx revealed that the expression of *GABRG3* was higher in several brain regions, such as the cerebellum and hypothalamus, as compared to other human tissues. The HBT database showed an increased *GABRG3* expression in the hippocampus and other brain regions during developmental stages. After birth, the expression of *GABRG3* was stabilized at a relatively high level in different regions of the brain throughout life (Fig. S5a). The dynamic expression of *GABRG3* in 11 areas of the neocortex was similar to the expression in other brain regions (Fig. S5b).

### Function analysis of the associated SNPs and sequenced variants

The function prediction revealed that the rs7180500, rs201602655 (p.Val233Met) and rs201427468 (p.Pro365Ser) in *GABRG3* might alter the regulatory motifs, as well as rs4906902 in *GABRB3* and rs4906771 in *ATP10A* (Table S8). Additionally, three programs (Polyphen2, SIFT and Mutation Taster) predicted that rs201602655 (p.Val233Met) in *GABRG3* might be deleterious and affect protein function (Table 2). The rs201427468 (p.Pro365Ser) in *GABRB3* was predicted to be damaging only by the Mutation Taster software.

## Discussion

Our study indicated that the C allele of rs7180500 in *GABRG3* was significantly associated with autism in 512 autism trios. The eQTL data from two web sources confirmed that this risk allele might be correlated with the expression of *GABRG3* in the cerebellum. Moreover, we sequenced the genes on chromosome 15q11-q13 region and identified two rare variants rs201602655 (p.Val233Met) and rs201427468 (p.Pro365Ser) in *GABRG3* and six rare variants in *GABRB3*. Among these mutations, the frequency of rs201602655 (p.Val233Met) in *GABRG3* in autistic children was significantly higher than that in healthy controls (9/512 vs. 2/575, Pearson *χ*^2^-test *χ*^2^ = 5.375, *p* = 0.020). The function prediction indicated that rs201602655 (p.Val233Met) might be deleterious. Additionally, rs4906902 in *GABRB3* and rs4906771 in *ATP10A* exhibited significant association with autism under the recessive model.

In the Psychiatry Genomics Consortium (PGC) ASD subset (available at: http://www.med.unc.edu/pgc/results-and-downloads), rs7180500 in *GABRG3* was nominally associated with autism (OR = 0.938; SE = 0.028; *p* = 0.021) (Table S5), whereas rs4906902 in *GABRB3* and rs4906771 in *ATP10A* did not show a significant association. Other significant risk alleles reported in Caucasians, such as rs7180158, rs7165604, rs12593579 and rs9806546 in *GABRB3*, did not exhibit a positive association with autism in our samples. Moreover, our study demonstrated that rs4906902 in *GABRB3* and rs4906771 in *ATP10A* were significantly associated with autism under the recessive model, although these positive results were not replicated in ASD subset of PGC. In addition, a previous study reported no significant association between GABA receptor genes on chromosome 15q11-q13 and autism in 166 Japanese autistic patients and 412 controls after Bonferroni correction. However, nominal significant associatin of rs3212337 in *GABRB3* and rs4887536 in *GABRG3* and autism were observed^46^. It is suggested that further search for susceptibility variants should be performed.

Our results were inconsistent with PGC autism data and previous study in Japanese population, which might result from a few reasons: First, ethnic heterogeneity might be considered. Due to different genetic backgrounds, the MAF of rs7180500 in our samples and the East Asian population was approximately 0.10, while 0.49 in Europeans. Second, ASD is a genetic heterogenous spectrum. Subjects of other studies were mostly ASD individuals. To decrease heterogeneity, our study recruited families with classical autistic individuals. Third, the genetic signal might be tagging other genetic variants, which directly contribute to the autism risk.

We detected a rare variant rs201602655 (p.Val233Met) in 9 of 512 autistic patients. The frequency of rs201602655 (p.Val233Met) in *GABRG3* in autistic children was significantly higher than that in healthy children. *GABRG3* had found to be intolerant to the heterozygous missense variant based on the *Z*-score of 1.92 from the Exome Aggregation Consortium (ExAC) database. This indicates that the detected missense variants might be deleterious. Among the 9 patients carrying rs201602655 (p.Val233Met), one patients carried the de novo variant, and the other 8 patients were inherited this variant from their parents (all heterozygotes). Previous studies showed that in the low-risk families, the de novo mutations might contribute to the development of autism. On the other hand, in high-risk families, the inherited variants might increase the risk and susceptibility to autism^47,48^. Heritability estimates strongly support a significant role for autosomal inherited factors^2,49^. Indeed, combined variants including rare de novo and inherited variants were reported to be affected in the case to reach the threshold for a fully penetrant phenotype, suggesting a 'multiple hit' model of ASD^50–52^. Our results might indicate that the rare variants detected in *GABRG3* might contribute to the increased risk of autism.

Autism is a complex disorder with high heritability and heterogeneity. Both common and rare variations contribute to liability. The common variants increase the risk for autism with a small effect, and the interaction with other susceptibility genes and environmental factors might underlie the pathogenesis of autism. Although the frequencies of rare variants were low, they might exhibit loss of function (LoF) effect that could be the causative factor for autism. Recent studies have identified distinct and individually rare genetic causes, suggesting that the genetic architecture of autism might contribute significantly to heterogeneous rare variants^28,48,53,54^. In this study, we detected that both common variant (rs7180500) and rare variant rs201602655 (p.Val233Met) in *GABRG3* were associated with autism in Chinese Han population. Our study provided new evidence for the contribution of common and rare variations to the etiology of autism. The potential risk of common variants and rare mutations in *GABRG3* remained to be explored in different ethnic populations using large sample studies.

Furthermore, the functional evidence indicated that abnormal expression of *GABRG3* and other GABA_A_ receptors subunits genes could serve as susceptibility factors for autism. Fatemi et al. further found an abnormal expression of *GABRG3* in the cerebellum and other Brodmann areas of autistic individuals^8^. The current study showed that the risk C allele of rs7180500 might lead to a lower expression of *GABRG3* in human cerebellum by eQTL data analysis, which might partially trigger abnormal social phenotypes. Several studies suggested that the dysregulation of GABAergic transmission and an imbalance between excitation and inhibition (E/I) in the selective neuronal circuits in the brain of autistic individuals might be involved in social and emotional processes^55,56^. GABA_A_ receptors, including *GABRG3*, played a critical role in modulating the intracellular Ca^2+^ concentration during different developmental stages. Along with the maturation of the central nervous system, the increase in intracellular Ca^2+^ concentration was gradually reduced via the regulation of GABA_A_ receptors^5^. Moreover, *GABRG3* contained benzodiazapine binding sites. Some pharmacological studies indicated that reduced benzodiazepine binding sites in GABA_A_ receptors were observed in several brain regions of autistic individuals^57–59^. Therefore, GABA_A_ receptor genes cluster including *GABRG*3 might play a role in the etiology of autism.

Our study has a few limitations. The other genes located on 15q11-13 need to be studied. The 15q13.3 microdeletion syndrome is associated with numerous conditions, including ASD, epilepsy, schizophrenia, and intellectual disability^60–64^. Deletion of genes located in this region, such as *CHRNA7* and *OTUD7A* was responsible for the 15q13.3 microdeletion syndrome^65,66^. Recent studies indicated that disruption of *OTUD7A* could cause neurodevelopmental deficits including abnormal cortical neuron morphology, which recapitulated some of the characteristics of the 15q13.3 microdeletion syndrome^67,68^. Therefore, genetic studies on the relationship of variants in candidate genes such as *CHRNA7* and *OTUD7A* on 15q11-q13 with autism should be performed.

Our study suggested that common variants and rare variants in *GABRG3* were significantly associated with autism. *GABRG3* might contribute to the pathogenesis of autism. Moreover, *GABRB3* and *ATP10A* located on chromosome 15q11-q13 might increase risk for autism in Chinese Han population.

## Electronic supplementary material

Supplementary Figures

Supplementary Tables
