# Supplementary material for: Association study and mutation sequencing of genes on chromosome 15q11-q13 identified GABRG3 as a susceptibility gene for autism in Chinese Han population
Source: Transl Psychiatry. 2018 Aug 14;8:152. doi: 10.1038/s41398-018-0197-4 (PMC6092396; doi:10.1038/s41398-018-0197-4)
Supplement: Supplementary file 1 — Supplementary Figures [file 41398_2018_197_MOESM1_ESM.docx]

**Association study and mutation sequencing in chromosome 15q11-q13 identified *GABRG3* as a susceptibility gene for autism in Chinese Han population**

Linyan Wang^1,2,3,4#^, Jun Li^1,2,3,4#^, Mei Shuang^1,2,3,4#^, Tianlan Lu^1,2,3,4^, Ziqi Wang^1,2,3,4^, Tian Zhang^1,2,3,4^, Weihua Yue^1,2,3,4^, Meixiang Jia^1,2,3,4^, Yanyan Ruan^1,2,3,4^, Jing Liu^1,2,3,4^，Zhiliu Wu^1,2,3,4,5^, Dai Zhang^1,2,3,4,6,7^, Lifang Wang^1,2,3,4^

^1^ Peking University Sixth Hospital, Beijing 100191, China.

^2^ Peking University Institute of Mental Health, Beijing 100191, China.

^3^ Key Laboratory of Mental Health, Ministry of Health (Peking University), Beijing 100191, China.

^4^ National Clinical Research Center for Mental Disorders, (Peking University Sixth Hospital), Beijing 100191, China.

^5^ The Affiliated Brain Hospital of Guangzhou Medical University (Guangzhou Huiai Hospital), Guangzhou, 510370, China.

^6^ Peking-Tsinghua Center for Life Sciences, Peking University, Beijing, 100871, China.

^7^ PKU-IDG/McGovern Institute for Brain Research, Peking University, Beijing 100871, China.

#These authors contributed equally to this work.

Correspondence author: Jing Liu (ljyuch@bjmu.edu.cn), Zhiliu Wu (email: zhiliu_wu@bjmu.edu.cn), Lifang Wang (email: lifangwang@bjmu.edu.cn)

Fax: 86-010-82021960

**Supplementary Materials**

**Figure S1.** **Schematic of sample processing by targeted sequencing.**

**Figure S2.** **Schematic of data analysis by targeted sequencing**.

**Figure S3. Six rare variants in *GABRB3* validated by Sanger sequencing.**

**Figure S4.** **eQTL effects of rs4906771 in *ATP10A* on 10 brain regions in the Braineac database.**

**Figure S5. Dynamic expression of *GABRG3* in human brain and 11 areas of neocortex.**

**Figure S1. Schematic of sample processing by targeted sequencing.**

**
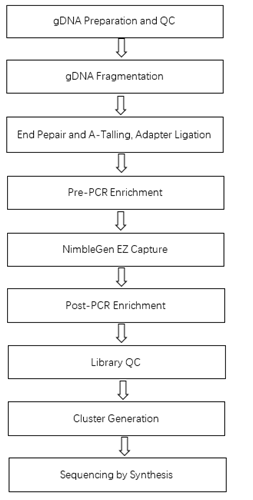
**

Abbreviations: QC: Quality controls; PCR: polymerase chain reaction.

**Figure S2. Schematic of data analysis by targeted sequencing.**

**
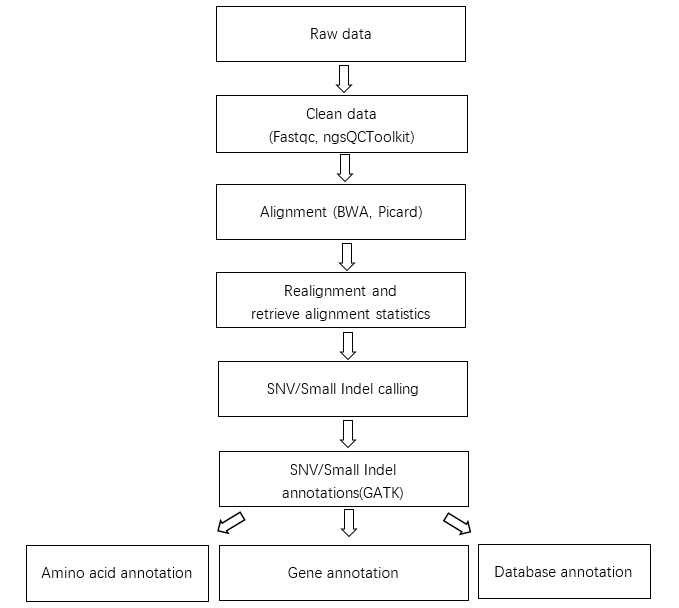
**

Abbreviations: QC: Quality controls; ngs: next generation sequencing; BWA: Burrows-Wheeler Aligner; GATK: Genome Analysis Toolkit.

**Figure S3.** **Six rare variants in *GABRB3* validated by Sanger sequencing.**


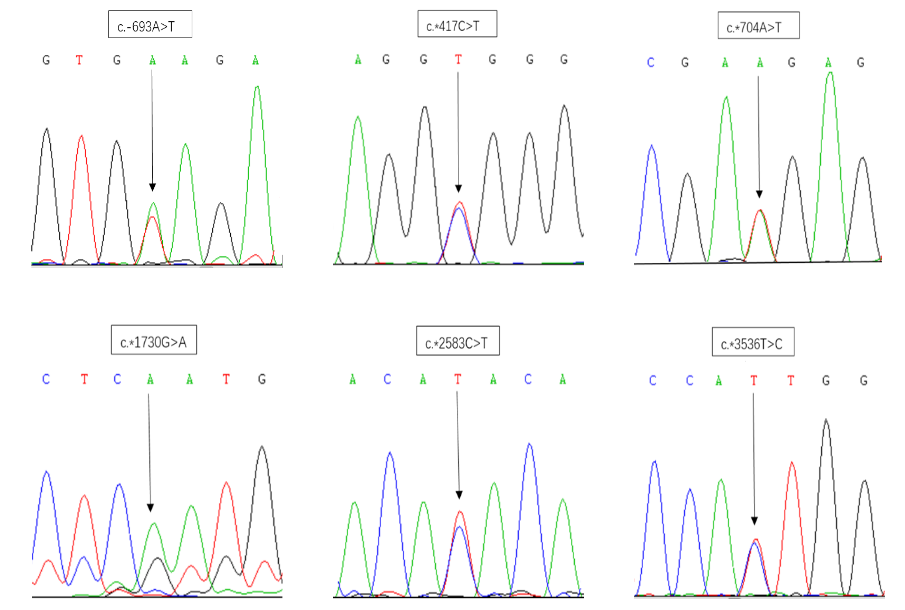


The position and alleles of each SNP are indicated with an arrow.

**Figure S4.** **eQTL effects of rs4906771 in *ATP10A* on 10 brain regions in the Braineac database.**

**
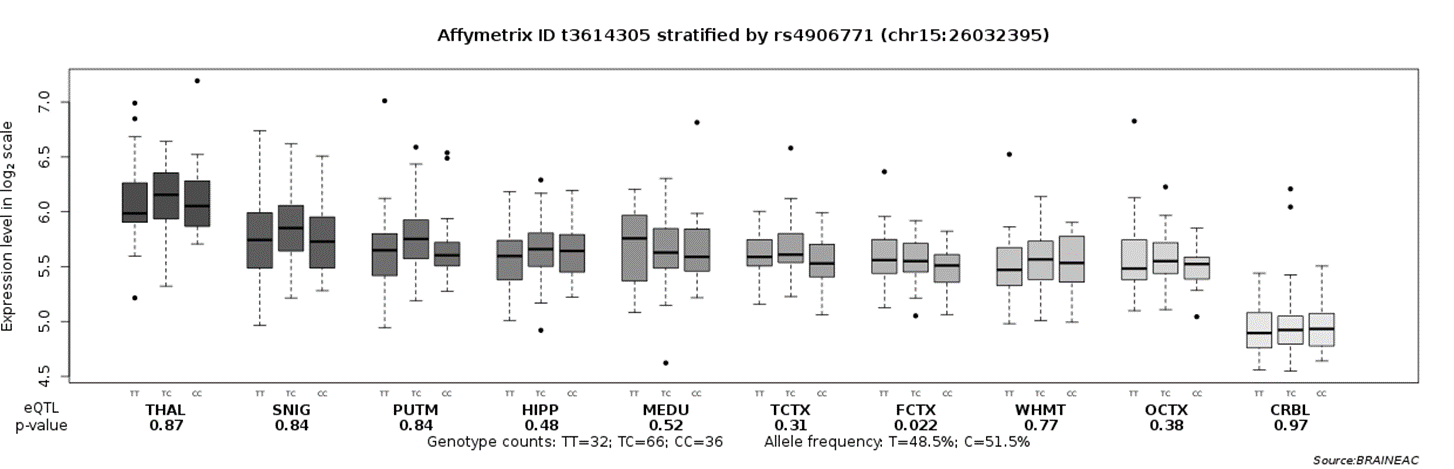
**

Abbreviations: WHMT: white matter; MEDU: medulla; PUTM: putamen; THAL: thalamus; SNIG: substantia nigra; HIPP: hippocampus; FCTX: frontal cortex; TCTX: temporal cortex; OCTX: occipital cortex; CRBL: cerebellum.

**Figure S5. Dynamic expression of *GABRG3* in human brain and 11 areas of neocortex.**


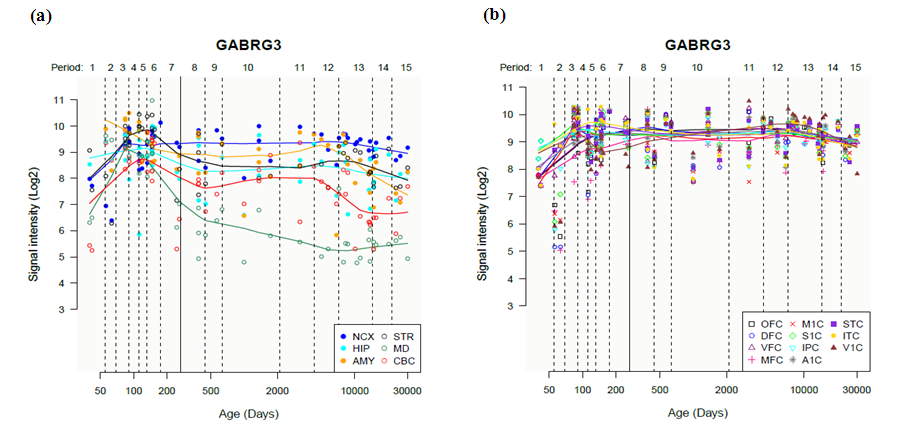


Dynamic expression of *GABRG3* in 6 brain regions (**a**) and 11 area of neocortex (**b**).

Abbreviation: NCX: neocortex; STR: striatum; HIP: hippocampus; MD: mediodorsal nucleus; AMY: amygdala; CBC: cerebellar cortex; OFC: the orbital cortices; M1C: the primary motor cortices; STC: the posterior superior cortices; DFC: the dorsolateral cortices; S1C: the primary somatosensory cortices; ITC: the anterior inferior cortices; VFC: the ventrolateral cortices; IPC: the posterior inferior cortices; V1C: the primary visual cortex; MFC: the media cortices; A1C: the primary auditory cortic.
