# Supplementary material for: Association study and mutation sequencing of genes on chromosome 15q11-q13 identified GABRG3 as a susceptibility gene for autism in Chinese Han population
Source: Transl Psychiatry. 2018 Aug 14;8:152. doi: 10.1038/s41398-018-0197-4 (PMC6092396; doi:10.1038/s41398-018-0197-4)
Supplement: Supplementary file 2 — Supplementary Tables [file 41398_2018_197_MOESM2_ESM.docx]

**Association study and mutation sequencing in chromosome 15q11-q13 identified *GABRG3* as a susceptibility gene for autism in Chinese Han population**

Linyan Wang^1,2,3,4#^, Jun Li^1,2,3,4#^, Mei Shuang^1,2,3,4#^, Tianlan Lu^1,2,3,4^, Ziqi Wang^1,2,3,4^, Tian Zhang^1,2,3,4^, Weihua Yue^1,2,3,4^, Meixiang Jia^1,2,3,4^, Yanyan Ruan^1,2,3,4^, Jing Liu^1,2,3,4^, Zhiliu Wu^1,2,3,4,5^, Dai Zhang^1,2,3,4,6,7^, Lifang Wang^1,2,3,4^

^1^ Peking University Sixth Hospital, Beijing 100191, China.

^2^ Peking University Institute of Mental Health, Beijing 100191, China.

^3^ Key Laboratory of Mental Health, Ministry of Health (Peking University), Beijing 100191, China.

^4^ National Clinical Research Center for Mental Disorders, (Peking University Sixth Hospital), Beijing 100191, China.

^5^ The Affiliated Brain Hospital of Guangzhou Medical University (Guangzhou Huiai Hospital), Guangzhou, 510370, China.

^6^ Peking-Tsinghua Center for Life Sciences, Peking University, Beijing, 100871, China.

^7^ PKU-IDG/McGovern Institute for Brain Research, Peking University, Beijing 100871, China.

#These authors contributed equally to this work.

Correspondence author: Jing Liu ([ljyuch@bjmu.edu.cn](mailto:ljyuch@bjmu.edu.cn)), Zhiliu Wu (email: zhiliu_wu@bjmu.edu.cn), Lifang Wang (email: lifangwang@bjmu.edu.cn)

Fax: 86-010-82021960

**Supplementary Materials**

**Table S1. DNA amplification and extend reaction primers of 41 tag SNPs for Agena Bioscience platform.**

**Table S2.** **Primers of the eight rare variants in *GABRG3* and *GABRB3* for Sanger sequencing.**

**Table S3.** **Information of 41 tag SNPs in chromosome 15q11-q13 and genotype frequencies in 512 autism trios of Chinese Han descent.**

**Table S4. Results of association analyses between 41 tag SNPs in chromosome 15q11-q13 in 512 trios by FBAT under a recessive model.**

**Table S5.** **Rare genetic variants of *GABRB3* detected in 512 autistic children.**

**Table S6. eQTL effects of rs4906771 in *ATP10A* on 10 brain regions in the Genotype-Tissue Expression (GTEx) database.**

**Table S7. Functional annotation for significantly associated SNPs and rare SNPs in *GABRG3*, *GABRB3* and *ATP10A*.**

**Table S8.** **The PGC ASD subset association results for 9 positive associated SNPs detected in 512 Chinese Han autism trios.**

**Table S1. DNA amplification and extend reaction primers of 41 tag SNPs for Agena Bioscience platform.**

| SNP ID | PCR primers | Extended primers |
| --- | --- | --- |
| rs10152278 | U: 5’ ACGTTGGATGTTGGCGCTAGAGTCAGCTAC 3’  L: 5’ ACGTTGGATGGCCCGGCTTGTTGTAGTTTA 3’ | 5’ GGAAACTAGACTATGGGATCA 3’ |
| rs12900552 | U: 5’ ACGTTGGATGTTATCTGACCCTGAGAGGAG 3’  L: 5’ ACGTTGGATGAGCCATTCAAAGACTCCCGA 3’ | 5’ ACTCCCGACTCTCAC 3’ |
| rs7177893 | U: 5’ ACGTTGGATGCTATGTGATGATCAATGTTGG 3’  L: 5’ ACGTTGGATGACAGTGACCAGTGCAATGAG 3’ | 5’ CATCGGCACCCCCCTTTCACTGCTAT 3’ |
| rs8025849 | U: 5’ ACGTTGGATGCTGTGTTACAATTGCCTGCG 3’  L: 5’ ACGTTGGATGATGACCCACCCAGGCCAGAT 3’ | 5’ ACACCTGTACAGCATGTGAC 3’ |
| rs8028189 | U: 5’ ACGTTGGATGTGCAGTGGGAACAGTGTAAG 3’  L: 5’ ACGTTGGATGACAAGATGTCAGGGACAATC 3’ | 5’ CTCATTTCTACACGCTGTAA 3’ |
| rs3812922 | U: 5’ ACGTTGGATGTCTGTAATCGGCGCGTTTTC 3’  L: 5’ ACGTTGGATGTGCAAACAGCTCCTTGATAG 3’ | 5’ CCCCAAGCTCCTTGATAGCAATGCC 3’ |
| rs3785 | U: 5’ ACGTTGGATGCTCCTTTGGGCCACTACATA 3’  L: 5’ ACGTTGGATGCTGCATATGTTTGCAGTTT 3’ | 5’ GCGGAATATGTTTGCAGTTTTCCATC 3’ |
| rs1009153 | U: 5’ ACGTTGGATGGGCTTAGGATATTGGTGCTG 3’  L: 5’ ACGTTGGATGAGTCTTGTGGAGGACTGTGC 3’ | 5’ GACTGTGCCCTCAGA 3’ |
| rs2289818 | U: 5’ ACGTTGGATGCTCACCAGGTTCAACAAGCA 3’  L: 5’ ACGTTGGATGAACAGAAATGGAGCTGCAGG 3’ | 5’ CTGCCTCAATTTCGTCGTA 3’ |
| rs2289823 | U: 5’ ACGTTGGATGAGAAGCTAGTGACACTGAGG 3’  L: 5’ ACGTTGGATGAGAAGCCATGGGGTGTTTTG 3’ | 5’ CATTGGGTGTTTTGGGTGGT 3’ |
| rs12441090 | U: 5’ ACGTTGGATGTGTGAAGAGGAAGAACTCGG 3’  L: 5’ ACGTTGGATGACAGGCCGGCACATGTCTTC 3’ | 5’ TCTTCCCACACACTGT 3’ |
| rs8025779 | U: 5’ ACGTTGGATGACCGGCACTATATCCCTACT 3’  L: 5’ ACGTTGGATGTGGAAGAACGAGTCACGCTG 3’ | 5’ TAGCAGGTGTGCACT 3’ |
| rs8042900 | U: 5’ ACGTTGGATGCTGACTCCACTCAGCGTTTC 3’  L: 5’ ACGTTGGATGGAAAACCTGATTTGAGCTCG 3’ | 5’ CTCGTGCCAAAAAACAATCT 3’ |
| rs8036523 | U: 5’ ACGTTGGATGTAGGAAGGCAAGTCAAGCAG 3’  L: 5’ ACGTTGGATGCCAAAAGCTATAGACATTGGC 3’ | 5’ CGACATTGGCTTGCATATTTGA 3’ |
| rs8037745 | U: 5’ ACGTTGGATGTGGAAATAGCATTTCCGTTG 3’  L: 5’ ACGTTGGATGAGCTGAATCTTTCATTCACC 3’ | 5’ ATTCACCTCTCCATCTAC 3’ |
| rs220030 | U: 5’ ACGTTGGATGTTTACACTCACCCTCAGGTC 3’  L: 5’ ACGTTGGATGCATTGATTGTGGTTATGGCG 3’ | 5’ TTACCTTTTCCAAACCAGCTTTTT 3’ |
| rs2732025 | U: 5’ ACGTTGGATGCCTGGTTCCTGGGAAGTAAC 3’  L: 5’ ACGTTGGATGAGGGACCCACCTTGAGTAAC 3’ | 5’ TAACAAACACATTTCTTTCACT 3’ |
| rs2732026 | U: 5’ ACGTTGGATGACTCTCGTCGCCTTTCTCAT 3’  L: 5’ ACGTTGGATGGACCTTGTAAGTGAAAGAAG 3’ | 5’ AAGCAGAAAATAAATCACTGCAGAGT 3’ |
| rs8179187 | U: 5’ ACGTTGGATGCTGCCTCCTTACAGTATGAC 3’  L: 5’ ACGTTGGATGTGATAACCCCATGTACCAGC 3’ | 5’ ACCAGCTGACTCATTT 3’ |
| rs2158306 | U: 5’ ACGTTGGATGATTCAGACCACAGCACTAGA 3’  L: 5’ ACGTTGGATGATAGAAGCCACAGGAGGAAC 3’ | 5’ TACTGATTTACCAATAACCAATTCA 3’ |
| rs12324063 | U: 5’ ACGTTGGATGAAGGAAGCCATAAGTCAGGG 3’  L: 5’ ACGTTGGATGGCAGGGCTGTGTTCCTTTTG 3’ | 5’ CCCATTTGCTTGTTCCAGCTTCTT 3’ |
| rs4906771 | U: 5’ ACGTTGGATGGTCTCTGCACTGCATAATTT 3’  L: 5’ ACGTTGGATGGATCTGGTAGCCTAAAGGAG 3’ | 5’ AGTGCTAAGCAAAAAAGTT 3’ |
| rs1345098 | U: 5’ ACGTTGGATGTCAAAGCGAGGGAAAATAAC 3’  L: 5’ ACGTTGGATGATTCTTACCAGCTCAGGAAC 3’ | 5’ GTGTGGGTGTATTTAGGG 3’ |
| rs11638520 | U: 5’ ACGTTGGATGACTGAGTTTTGTTCTATGGC 3’  L: 5’ ACGTTGGATGTCTTCTTAAGTGCACGTGGC 3’ | 5’ AGTGGCGTGTCCTGTA 3’ |
| rs73356452 | U: 5’ ACGTTGGATGAAATCAGCAAGCTGGTTCCC 3’  L: 5’ ACGTTGGATGCTCCTGGTGCTTACAAAGCC 3’ | 5’ CCCTAAAGCCAAATGCTGATTC 3’ |
| rs1511488 | U: 5’ ACGTTGGATGAGATCTTCTCATCTCACGCC 3’  L: 5’ ACGTTGGATGTGGAAAGGACAAGTCTCTGG 3’ | 5’ GGATCTGGATTTGCTCCAA 3’ |
| rs12438141 | U: 5’ ACGTTGGATGATACATCCCAGGGAAGTCAC 3’  L: 5’ ACGTTGGATGCCATCTCTTTGTGACTGTGC 3’ | 5’ CCCCCGACTGTGCTCTTCTCTTT 3’ |
| rs9806546 | U: 5’ ACGTTGGATGGGACCTCATATGAGTGGAGT 3’  L: 5’ ACGTTGGATGGACATGTTTCCACATGGATG 3’ | 5’ GCAGTCACAAAGGACAAA 3’ |
| rs1863467 | U: 5’ ACGTTGGATGCCAGTATGGTTGAACTCCAG 3’  L: 5’ ACGTTGGATGATGAGAGCCATAGGTGTGTC 3’ | 5’ GGGCGGTGTGTCACCGATG 3’ |
| rs7180158 | U: 5’ ACGTTGGATGAGAGGAAACAGCAGGCCTTC 3’  L: 5’ ACGTTGGATGGTGAACATCACACTCAATGC 3’ | 5’ AGTTTTCTTCTAAGATCAGGAAC 3’ |
| rs12593579 | U: 5’ ACGTTGGATGGAGACAGAGTCTCGCTATGG 3’  L: 5’ ACGTTGGATGGTTGCAGTGTGCTGAGATCC 3’ | 5’ TGCTGAGATCCTACAAC 3’ |
| rs7165604 | U: 5’ ACGTTGGATGCAGTGACAGAAAGATAACAC 3’  L: 5’ ACGTTGGATGCTCTTAGACTTATGGATTAC 3’ | 5’ GATTACTTAAAAGTTTACTGTTTAAC 3’ |
| rs8041610 | U: 5’ ACGTTGGATGAGGCTCTGCCCTAATTGTGG 3’  L: 5’ ACGTTGGATGTTGGAGTTACTGCCAGGTGC 3’ | 5’ TCTCCCCACTGATACACACTGAAGGC 3’ |
| rs4906902 | U: 5’ ACGTTGGATGCTGTTCCAAAAATAAAATAGC 3’  L: 5’ ACGTTGGATGCTCACGTTGGCATGTTTCTG 3’ | 5’ GCATGTTTCTGTGCATT 3’ |
| rs35586628 | U: 5’ ACGTTGGATGGGCTATGCAAACTACTGGTG 3’  L: 5’ ACGTTGGATGACTGAAGTGCTCAACCACAG 3’ | 5’ GAACGGGTGATTCGTGCTTT 3’ |
| rs7403021 | U: 5’ ACGTTGGATGACCGAAAATGGCATTTACGC 3’  L: 5’ ACGTTGGATGCACTGTTGCGATTGCAATAC 3’ | 5’ CACTTTGCAAGACTAACTTTCTGAA 3’ |
| rs7180500 | U: 5’ ACGTTGGATGCCATCTTCTCTTTCTGGAGC 3’  L: 5’ ACGTTGGATGTTCAATACCAGTTACCTGTC 3’ | 5’ CACTAGCTGTAGTATGTTTAGTAA 3’ |
| rs12910555 | U: 5’ ACGTTGGATGTAGCTGTGGGAACCTGAACA 3’  L: 5’ ACGTTGGATGACCAACCCTGTGATAGAAGC 3’ | 5’ AAAGAAAGTGAAGCAAAGAA 3’ |
| rs897173 | U: 5’ ACGTTGGATGCACTGCCAGAATGATAGATG 3’  L: 5’ ACGTTGGATGGATTATTTTCACATCCCTGC 3’ | 5’ ACATCCCTGCAATGAA 3’ |
| rs4778159 | U: 5’ ACGTTGGATGCTCTTGATTCATGTAACAAA 3’  L: 5’ ACGTTGGATGGTGCCTTTCTCAGAGGAATG 3’ | 5’ TGTAGTTTTTGGTCTTCCTTC 3’ |
| rs140679 | U: 5’ ACGTTGGATGAGTAGTTGAGGGTGGCATAC 3’  L: 5’ ACGTTGGATGGTGACCGCCATGGACCTTTT 3’ | 5’ GGACATGGACCTTTTTGTGAC 3’ |

Abbreviations: U, upper primer; L, Lower primer.

All primers were designed according to the forward sequence of DNA provided by release of hg19 assembly.

**Table S2**. **Primers of the eight rare variants in *GABRG3* and *GABRB3* for Sanger sequencing.**

| Gene | Variants | Forward | Reverse | Size (bp) |
| --- | --- | --- | --- | --- |
| *GABRG3* | rs201602655 | GACACCACTCCCTGTGTGTT | CAGAACTCTCCCCATCAGCA | 710 |
|  | rs201427468 | ACGTGGCTTGGTTTAGTCAT | CATGTGCATCACTCATTGGTCT | 283 |
| *GABRB3* | c.-693A>T | TCAGGTACTGCGGTCACATTTT | ATTAGAAGGCTACTGGCGCAC | 503 |
|  | c.*417C>T | TGGTCAGAGCACCCATTCTC | CAGCACTCCTTCCGATGATCC | 501 |
|  | c.*704A>T | AGTGCCCATGTGTATGGAAGAC | AGACGTCTATGCTTTCTGTTGGA | 694 |
|  | c.*1730G>A | CTGCAGGAACATGACCCGTA | GGGATAGTCCACACCACACG | 529 |
|  | c.*2583C>T | GCAGAAGAGGGACCCCAAAAT | CCAACAACGTCAGAGGTCCG | 680 |
|  | c.*3536T>C | TTTCCAAAATGCGCCACAGG | GGCGCCTTTCAATGCAGTAG | 839 |

All primers were designed according to the forward sequence of DNA provided by release of hg19 assembly.

**Table S3.** **Information of 41 tag SNPs in chromosome 15q11-q13 and genotype frequencies in 512 autism trios of Chinese Han descent.**

| Gene | Marker | Chromosome | Genotype frequencies in children | | | *p* _HWE_ ^a^ | Genotype frequencies in parents | | | *p* _HWE_ ^b^ |
| --- | --- | --- | --- | --- | --- | --- | --- | --- | --- | --- |
| *NIPA1* | rs10152278 | 15:22786139 | G G | G A | A A |  | G G | G A | A A |  |
|  |  |  | 168 | 254 | 88 | 0.63 | 335 | 506 | 182 | 0.70 |
|  | rs12900552 | 15:22795211 | A A | A G | G G |  | A A | A G | G G |  |
|  |  |  | 168 | 246 | 94 | 0.81 | 339 | 512 | 171 | 0.34 |
|  | rs7177893 | 15:22807275 | G G | G T | T T |  | G G | G T | T T |  |
|  |  |  | 403 | 96 | 6 | 0.92 | 805 | 194 | 19 | 0.07 |
|  | rs8025849 | 15:22825211 | A A | A G | G G |  | A A | A G | G G |  |
|  |  |  | 337 | 161 | 11 | 0.10 | 684 | 300 | 38 | 0.48 |
| *NIPA2* | rs8028189 | 15:22849067 | G G | G C | C C |  | G G | G C | C C |  |
|  |  |  | 259 | 208 | 43 | 0.89 | 537 | 424 | 62 | 0.07 |
|  | rs3812922 | 15:22866361 | C C | C A | A A |  | C C | C A | A A |  |
|  |  |  | 318 | 166 | 22 | 0.95 | 607 | 355 | 54 | 0.82 |
|  | rs3785 | 15:22867866 | G G | G A | A A |  | G G | G A | A A |  |
|  |  |  | 188 | 202 | 65 | 0.37 | 350 | 445 | 124 | 0.21 |
| *CYFIP1* | rs1009153 | 15:22896157 | A A | A G | G G |  | A A | A G | G G |  |
|  |  |  | 136 | 261 | 111 | 0.50 | 306 | 503 | 215 | 0.75 |
|  | rs2289818 | 15:22912200 | G G | G C | C C |  | G G | G C | C C |  |
|  |  |  | 177 | 253 | 74 | 0.28 | 351 | 478 | 185 | 0.32 |
|  | rs2289823 | 15:22945116 | C C | C T | T T |  | C C | C T | T T |  |
|  |  |  | 369 | 84 | 6 | 0.63 | 749 | 148 | 12 | 0.13 |
|  | rs12441090 | 15:22967498 | G G | G A | A A |  | G G | G A | A A |  |
|  |  |  | 269 | 195 | 45 | 0.26 | 532 | 406 | 83 | 0.65 |
|  | rs8025779 | 15:22979151 | C C | C G | G G |  | C C | C G | G G |  |
|  |  |  | 208 | 238 | 63 | 0.69 | 428 | 472 | 120 | 0.56 |
|  | rs8042900 | 15:22980985 | A A | A G | G G |  | A A | A G | G G |  |
|  |  |  | 351 | 134 | 23 | 0.03 | 698 | 293 | 31 | 0.97 |
| *SNRPN* | rs8036523 | 15:24824408 | T T | T G | G G |  | T T | T G | G G |  |
|  |  |  | 301 | 178 | 29 | 0.69 | 604 | 362 | 54 | 0.98 |
|  | rs8037745 | 15:24902158 | A A | A G | G G |  | A A | A G | G G |  |
|  |  |  | 369 | 131 | 7 | 0.22 | 716 | 273 | 30 | 0.52 |
| *SNURF* | rs220030 | 15:24954621 | C C | C T | T T |  | C C | C T | T T |  |
|  |  |  | 119 | 267 | 118 | 0.18 | 277 | 507 | 234 | 0.95 |
|  | rs2732025 | 15:24966663 | T T | T G | G G |  | T T | T G | G G |  |
|  |  |  | 118 | 274 | 118 | 0.09 | 270 | 519 | 235 | 0.63 |
|  | rs2732026 | 15:24971341 | A A | A C | C C |  | A A | A C | C C |  |
|  |  |  | 142 | 260 | 101 | 0.36 | 291 | 503 | 220 | 0.92 |
| *UBE3A* | rs8179187 | 15:25407179 | T T | T G | G G |  | T T | T G | G G |  |
|  |  |  | 204 | 238 | 68 | 0.91 | 405 | 489 | 129 | 0.32 |
|  | rs2158306 | 15:25435414 | C C | C T | T T |  | C C | C T | T T |  |
|  |  |  | 199 | 233 | 73 | 0.72 | 406 | 475 | 132 | 0.71 |
| *ATP10A* | rs12324063 | 15:25743809 | G G | G A | A A |  | G G | G A | A A |  |
|  |  |  | 190 | 224 | 81 | 0.28 | 345 | 484 | 156 | 0.52 |
|  | rs4906771 | 15:25787248 | C C | C T | T T |  | C C | C T | T T |  |
|  |  |  | 118 | 276 | 110 | 0.03 | 235 | 502 | 278 | 0.77 |
|  | rs1345098 | 15:25812475 | T T | T G | G G |  | T T | T G | G G |  |
|  |  |  | 321 | 173 | 15 | 0.15 | 635 | 333 | 47 | 0.69 |
|  | rs11638520 | 15:25828376 | T T | T G | G G |  | T T | T G | G G |  |
|  |  |  | 163 | 224 | 118 | 0.02 | 289 | 497 | 230 | 0.56 |
|  | rs73356452 | 15:25865066 | C C | C T | T T |  | C C | C T | T T |  |
|  |  |  | 396 | 106 | 7 | 0.98 | 798 | 208 | 13 | 0.89 |
|  | rs1511488 | 15:25866774 | G G | G C | C C |  | G G | G C | C C |  |
|  |  |  | 436 | 69 | 2 | 0.67 | 870 | 137 | 8 | 0.31 |
| *GABRB3* | rs12438141 | 15:26625455 | C C | C T | T T |  | C C | C T | T T |  |
|  |  |  | 437 | 65 | 5 | 0.15 | 856 | 153 | 5 | 0.51 |
|  | rs9806546 | 15:26648239 | A A | A G | G G |  | A A | A G | G G |  |
|  |  |  | 358 | 137 | 14 | 0.84 | 704 | 286 | 26 | 0.63 |
|  | rs1863467 | 15:26688587 | T T | C T | C C |  | T T | C T | C C |  |
|  |  |  | 96 | 220 | 192 | 0.02 | 205 | 468 | 340 | 0.06 |
|  | rs7180158 | 15:26733091 | A A | A G | G G |  | A A | A G | G G |  |
|  |  |  | 90 | 213 | 201 | 0.01 | 152 | 451 | 413 | 0.11 |
|  | rs12593579 | 15:26742985 | A A | A C | C C |  | A A | A C | C C |  |
|  |  |  | 243 | 214 | 51 | 0.70 | 477 | 448 | 97 | 0.32 |
|  | rs7165604 | 15:26749309 | T T | T C | C C |  | T T | T C | C C |  |
|  |  |  | 221 | 209 | 54 | 0.66 | 463 | 420 | 118 | 0.13 |
|  | rs8041610 | 15:26763117 | A A | A C | C C |  | A A | A C | C C |  |
|  |  |  | 169 | 255 | 86 | 0.54 | 343 | 510 | 166 | 0.31 |
|  | rs4906902 | 15:26774621 | A A | A G | G G |  | A A | A G | G G |  |
|  |  |  | 245 | 200 | 60 | 0.06 | 424 | 477 | 108 | 0.12 |
| *GABRA5* | rs35586628 | 15:26886993 | T T | T C | C C |  | T T | T C | C C |  |
|  |  |  | 183 | 242 | 75 | 0.73 | 350 | 499 | 168 | 0.66 |
| *GABRG3* | rs7403021 | 15:26970515 | C C | C T | T T |  | C C | C T | T T |  |
|  |  |  | 326 | 163 | 13 | 0.16 | 667 | 319 | 25 | 0.07 |
|  | rs7180500 | 15:27008032 | C C | C A | A A |  | C C | C A | A A |  |
|  |  |  | 418 | 80 | 1 | 0.15 | 816 | 190 | 7 | 0.25 |
|  | rs12910555 | 15:27122424 | A A | A G | G G |  | A A | A G | G G |  |
|  |  |  | 388 | 114 | 7 | 0.67 | 773 | 223 | 23 | 0.15 |
|  | rs897173 | 15:27224754 | A A | A G | G G |  | A A | A G | G G |  |
|  |  |  | 183 | 246 | 81 | 0.91 | 345 | 498 | 181 | 0.95 |
|  | rs4778159 | 15:27396841 | A A | A T | T T |  | A A | A T | T T |  |
|  |  |  | 250 | 202 | 52 | 0.24 | 472 | 416 | 119 | 0.07 |
|  | rs140679 | 15:27527530 | T T | T C | C C |  | T T | T C | C C |  |
|  |  |  | 247 | 198 | 58 | 0.06 | 485 | 429 | 106 | 0.44 |

^a^ Hardy-Weinberg equilibrium *p* value for genotype distributions in children affected with autism.

^b^ Hardy-Weinberg equilibrium *p* value for genotype distributions in parents.

**Table S4. Results of association analyses between 41 tag SNPs in chromosome 15q11-q13 in 512 trios by FBAT under a recessive model.**

| Gene symbol | Marker | Chromosome | Allele | Afreq | Fam | T : U^a^ | S-E (S) | Var (S) | Z | *p*^b^ |
| --- | --- | --- | --- | --- | --- | --- | --- | --- | --- | --- |
| *NIPA1* | rs10152278 | 15:22786139 | G | 0.571 | 202 | 259 : 239 | -7.00 | 43.250 | -1.064 | 0.287 |
|  |  |  | A | 0.429 | 296 | 239 : 259 | 3.00 | 66.750 | 0.367 | 0.713 |
|  | rs12900552 | 15:22795211 | G | 0.419 | 290 | 263 : 239 | -10.50 | 64.125 | -1.311 | 0.190 |
|  |  |  | A | 0.581 | 216 | 239 : 263 | 3.50 | 45.625 | 0.518 | 0.604 |
|  | rs7177893 | 15:22807275 | G | 0.887 | 23 | 105 : 85 | -1.50 | 4.500 | -0.707 | 0.480 |
|  |  |  | T | 0.113 | 167 | 85 : 105 | 8.50 | 40.500 | 1.336 | 0.180 |
|  | rs8025849 | 15:22825211 | A | 0.820 | 51 | 155 : 139 | -6.50 | 10.250 | -2.030 | 0.042 |
|  |  |  | G | 0.180 | 245 | 139 : 155 | 0.50 | 58.750 | 0.065 | 0.948 |
| *NIPA2* | rs8028189 | 15:22849067 | C | 0.273 | 293 | 220 : 196 | -14.00 | 67.125 | -1.709 | 0.070 |
|  |  |  | G | 0.727 | 123 | 196 : 220 | -2.00 | 24.625 | -0.403 | 0.687 |
|  | rs3812922 | 15:22866361 | C | 0.780 | 78 | 198 : 152 | -2.50 | 15.625 | -0.632 | 0.527 |
|  |  |  | A | 0.220 | 273 | 152 : 198 | 20.00 | 64.375 | 2.493 | 0.013 |
|  | rs3785 | 15:22867866 | G | 0.628 | 131 | 190 : 170 | -0.75 | 26.563 | -0.146 | 0.884 |
|  |  |  | A | 0.372 | 229 | 170 : 190 | 9.25 | 51.063 | 1.294 | 0.196 |
| *CYFIP1* | rs1009153 | 15:22896157 | G | 0.456 | 280 | 267 : 229 | -15.50 | 62.125 | -1.967 | 0.049 |
|  |  |  | A | 0.544 | 219 | 229 : 267 | 4.00 | 46.875 | 0.584 | 0.559 |
|  | rs2289818 | 15:22912200 | G | 0.588 | 197 | 254 : 211 | -14.00 | 42.125 | -2.157 | 0.031 |
|  |  |  | C | 0.412 | 270 | 211 : 254 | 6.50 | 60.375 | 0.837 | 0.403 |
|  | rs2289823 | 15:22945116 | C | 0.907 | 10 | 67 : 55 | -1.50 | 1.875 | -1.095 | 0.273 |
|  |  |  | T | 0.093 | 113 | 55 : 67 | 5.00 | 27.625 | 0.951 | 0.341 |
|  | rs12441090 | 15:22967498 | G | 0.720 | 114 | 205 : 191 | 0.75 | 23.563 | 0.155 | 0.877 |
|  |  |  | A | 0.280 | 283 | 191 : 205 | 7.25 | 65.813 | 0.894 | 0.371 |
|  | rs8025779 | 15:22979151 | C | 0.650 | 162 | 238 : 220 | -10.00 | 33.500 | -1.728 | 0.084 |
|  |  |  | G | 0.350 | 297 | 220 : 238 | -1.50 | 67.250 | -0.183 | 0.859 |
|  | rs8042900 | 15:22980985 | G | 0.169 | 237 | 145 : 142 | 3.00 | 56.625 | 1.432 | 0.152 |
|  |  |  | A | 0.831 | 50 | 142 : 145 | 4.50 | 9.875 | 0.399 | 0.690 |
| *SNRPN* | rs8036523 | 15:24824408 | G | 0.227 | 280 | 179 : 177 | 2.25 | 66.438 | 0.276 | 0.782 |
|  |  |  | T | 0.773 | 77 | 177 : 179 | 2.75 | 15.688 | 0.694 | 0.487 |
|  | rs8037745 | 15:24902158 | A | 0.847 | 43 | 155 : 109 | -8.00 | 8.375 | -2.764 | 0.006 |
|  |  |  | G | 0.153 | 221 | 109 : 155 | 15.00 | 52.875 | 2.063 | 0.039 |
| *SNURF* | rs220030 | 15:24954621 | T | 0.487 | 256 | 254 : 236 | -11.25 | 56.813 | -1.493 | 0.135 |
|  |  |  | C | 0.513 | 235 | 236 : 254 | -2.75 | 51.563 | -0.383 | 0.702 |
|  | rs2732025 | 15:24966663 | G | 0.485 | 267 | 264 : 246 | -12.25 | 59.188 | -1.592 | 0.111 |
|  |  |  | T | 0.514 | 244 | 246 : 264 | -3.75 | 53.438 | -0.513 | 0.607 |
|  | rs2732026 | 15:24971341 | A | 0.536 | 220 | 245 : 235 | -9.25 | 48.063 | -1.334 | 0.182 |
|  |  |  | C | 0.464 | 260 | 235 : 245 | -4.25 | 58.063 | -0.558 | 0.577 |
| *UBE3A* | rs8179187 | 15:25407179 | T | 0.635 | 173 | 242 : 237 | -4.25 | 35.938 | -0.709 | 0.478 |
|  |  |  | G | 0.365 | 307 | 237 : 242 | -2.25 | 69.438 | -0.27 | 0.787 |
|  | rs2158306 | 15:25435414 | T | 0.366 | 283 | 232 : 227 | -3.25 | 63.188 | -0.409 | 0.683 |
|  |  |  | C | 0.634 | 176 | 227 : 232 | -0.75 | 36.438 | -0.124 | 0.901 |
| *ATP10A* | rs12324063 | 15:25743809 | G | 0.601 | 185 | 240 : 207 | -4.75 | 39.313 | -0.758 | 0.449 |
|  |  |  | A | 0.399 | 263 | 207 : 240 | 11.25 | 58.813 | 1.467 | 0.142 |
|  | rs4906771 | 15:25787248 | C | 0.487 | 252 | 270 : 212 | -25.25 | 55.313 | -3.395 | **0.00069** |
|  |  |  | T | 0.513 | 234 | 212 : 270 | 2.75 | 50.813 | 0.386 | 0.700 |
|  | rs1345098 | 15:25812475 | T | 0.796 | 58 | 181 : 141 | -5.25 | 11.813 | 1.528 | 0.126 |
|  |  |  | G | 0.204 | 267 | 141 : 181 | 13.25 | 64.063 | -1.655 | 0.098 |
|  | rs11638520 | 15:25828376 | T | 0.532 | 223 | 250 : 223 | -2.00 | 48.625 | -0.287 | 0.774 |
|  |  |  | G | 0.468 | 252 | 223 : 250 | 10.50 | 55.875 | 1.405 | 0.160 |
|  | rs73356452 | 15:25865066 | C | 0.883 | 27 | 102 : 100 | -1.25 | 5.188 | -0.549 | 0.583 |
|  |  |  | T | 0.117 | 177 | 100 : 102 | -1.25 | 42.688 | -0.191 | 0.848 |
|  | rs1511488 | 15:25866774 | G | 0.927 | n/a | 73 : 59 | n/a | n/a | n/a | n/a |
|  |  |  | C | 0.073 | 124 | 59 : 73 | 6.75 | 30.563 | 1.221 | 0.222 |
| *GABRB3* | rs12438141 | 15:26625455 | C | 0.922 | 15 | 86 : 60 | 0.25 | 2.813 | 0.149 | 0.881 |
|  |  |  | T | 0.078 | 131 | 60 : 86 | 13.25 | 31.813 | 2.349 | 0.019 |
|  | rs9806546 | 15:26648239 | A | 0.833 | 53 | 150 : 133 | -3.00 | 10.625 | -0.920 | 0.357 |
|  |  |  | G | 0.167 | 231 | 133 : 150 | 5.00 | 55.125 | 0.673 | 0.501 |
|  | rs1863467 | 15:26688587 | C | 0.574 | 197 | 253 : 202 | -5.75 | 42.313 | -0.884 | 0.377 |
|  |  |  | T | 0.426 | 260 | 202 : 253 | 18.75 | 58.063 | 2.461 | 0.014 |
|  | rs7180158 | 15:26733091 | A | 0.359 | 280 | 224 : 209 | -1.00 | 64.250 | -0.125 | 0.900 |
|  |  |  | G | 0.641 | 153 | 209 : 224 | 6.50 | 32.500 | 1.140 | 0.254 |
|  | rs12593579 | 15:26742985 | A | 0.689 | 138 | 224 : 215 | 1.50 | 28.875 | 0.279 | 0.780 |
|  |  |  | C | 0.311 | 302 | 215 : 224 | 6.50 | 69.875 | 0.778 | 0.439 |
|  | rs7165604 | 15:26749309 | T | 0.684 | 135 | 192 : 186 | -8.50 | 28.000 | -1.606 | 0.108 |
|  |  |  | C | 0.316 | 243 | 186 : 192 | -5.50 | 55.000 | -0.742 | 0.458 |
|  | rs8041610 | 15:26763117 | C | 0.414 | 289 | 253 : 246 | -7.50 | 64.500 | -0.934 | 0.350 |
|  |  |  | A | 0.586 | 213 | 246 : 253 | -4.50 | 45.500 | -0.667 | 0.504 |
|  | rs4906902 | 15:26774621 | A | 0.667 | 161 | 263 : 197 | -5.00 | 32.875 | -0.872 | 0.383 |
|  |  |  | G | 0.333 | 299 | 197 : 263 | 28.00 | 67.375 | 3.441 | **0.00065** |
| *GABRA5* | rs35586628 | 15:26886993 | T | 0.595 | 197 | 254 : 226 | -7.00 | 42.125 | -1.079 | 0.281 |
|  |  |  | C | 0.405 | 283 | 226 : 254 | 7.00 | 63.625 | 0.878 | 0.380 |
| *GABRG3* | rs7403021 | 15:26970515 | C | 0.815 | 63 | 158 : 147 | -7.25 | 12.438 | -2.056 | 0.040 |
|  |  |  | T | 0.185 | 242 | 147 : 158 | -1.75 | 57.188 | -0.231 | 0.817 |
|  | rs7180500 | 15:27008032 | C | 0.904 | 21 | 112 : 66 | -5.75 | 4.063 | -2.853 | 0.0043 |
|  |  |  | A | 0.096 | 157 | 66 : 112 | 17.25 | 38.063 | 2.796 | 0.0052 |
|  | rs12910555 | 15:27122424 | A | 0.872 | 31 | 120 : 102 | -1.50 | 6.000 | -0.612 | 0.540 |
|  |  |  | G | 0.128 | 192 | 102 : 120 | 7.00 | 46.250 | 1.029 | 0.303 |
|  | rs897173 | 15:27224754 | A | 0.581 | 216 | 263 : 229 | -7.25 | 46.313 | -1.065 | 0.287 |
|  |  |  | G | 0.419 | 278 | 229 : 263 | 9.75 | 61.813 | 1.240 | 0.215 |
|  | rs4778159 | 15:27396841 | A | 0.684 | 132 | 217 : 177 | -4.75 | 28.438 | -0.891 | 0.373 |
|  |  |  | T | 0.316 | 263 | 177 : 217 | 14.75 | 61.188 | 1.886 | 0.059 |
|  | rs140679 | 15:27527530 | T | 0.687 | 144 | 214 : 199 | 2.25 | 30.188 | 0.410 | 0.682 |
|  |  |  | C | 0.313 | 271 | 199 : 214 | 8.75 | 61.938 | 1.112 | 0.266 |

Abbreviations: Afreq, allele frequency; Fam, number of informative families; S, test statistics for the observed number of transmitted alleles; E(S), expected value of S under the null hypothesis (i.e., no linkage and no association); n/a, not applicable.

^a^ The ratio of trasmisson to untransmisso (T∶U) for each selected SNP was calculated by the Haploveiw version 4.2.

^b^ *p* value with bold character means the statistical significance persists even after the Bonferroni correction.

**Table S5.** **Rare genetic variants of *GABRB3* detected in 512 autistic children.**

| Gene | Nucleotide change | Location | Position | MAF | Variants origin |
| --- | --- | --- | --- | --- | --- |
| *GABRB3* | c.-693A>T | 5'UTR | 15:27019565 | 0.002 | 1 (maternal) |
|  | c.*417C>T | 3'UTR | 15:26825563 | 0.002 | 1 (paternal) |
|  | c.*704A>T | 3'UTR | 15: 26792236 | 0.002 | 1 (maternal) |
|  | c.*1730G>A | 3'UTR | 15: 26791210 | 0.002 | 1 (paternal) |
|  | c.*2583C>T | 3'UTR | 15: 26790357 | 0.002 | 1 (paternal) |
|  | c.*3536T>C | 3'UTR | 15: 26789404 | 0.002 | 1 (maternal) |

Abbreviations: MAF, minor allele frequency.

**Table S6. eQTL effects of rs4906771 in *ATP10A* on 10 brain regions in the Genotype-Tissue Expression (GTEx) database.**

| Gene symbol | SNP ID | *p* ^a^ | Effect size | T-Statistic | Standard Error | Tissue |
| --- | --- | --- | --- | --- | --- | --- |
| *ATP10A* | rs4906771 | 0.240 | -0.140 | -1.20 | 0.120 | Cerebellar Hemisphere |
|  |  | 0.340 | 0.098 | 0.95 | 0.100 | Cortex |
|  |  | 0.120 | -0.160 | -1.60 | 0.100 | Frontal Cortex |
|  |  | **0.043** | 0.230 | 2.10 | 0.110 | Hippocampus |
|  |  | 0.460 | -0.075 | -0.74 | 0.100 | Hypothalamus |
|  |  | 0.310 | -0.110 | -1.00 | 0.110 | Nucleus accumbens (basal ganglia) |
|  |  | 0.750 | -0.024 | -0.32 | 0.077 | Putamen (basal ganglia) |
|  |  | 0.410 | -0.110 | -0.82 | 0.130 | Anterior cingulate cortex |
|  |  | 0.510 | -0.056 | -0.66 | 0.086 | Caudate (basal ganglia) |
|  |  | 0.170 | -0.150 | -1.40 | 0.110 | Cerebellum |

^a^ *p* value below 0.05 was noted with bold character.

**Table S7.** **Functional annotation for significantly associated SNPs and rare SNPs in *GABRG3*, *GABRB3* and *ATP10A*.**

| Gene | Variant | Function annotation | LD  (*r*^2^) | LD  (*D*') | Promoter histone marks | Enhancer histone  marks | DNAse | Motifs changed |
| --- | --- | --- | --- | --- | --- | --- | --- | --- |
| *GABRG3* | rs7180500 | intronic | 1 | 1 | n/a | n/a | BRN | HDAC2, Irf, p300 |
|  | rs201602655 | missense | 1 | 1 | n/a | BRST, SKIN | SKIN, SKIN | Myc, Nrf1 |
|  | rs201427468 | missense | 1 | 1 | n/a | n/a | BLD | HNF6, Pbx3 |
| *GABRB3* | rs4906902 | n/a | 1 | 1 | IPSC | ESC, IPSC, BRN | n/a | 7 altered motifs |
| *ATP10A* | rs4906771 | intronic | 1 | 1 | n/a | 9 tissues | n/a | 5 altered motifs |

Abbreviations: n/a, not applicable.

Note: The informations were from the HaploReg database.

**Table S8. The PGC ASD subset association results for 9 positive associated SNPs detected in 512 Chinese Han autism trios.**

| Gene | SNP ID | Chromosome | Ref | Alt | Freq | OR | SE | *p^a^* |
| --- | --- | --- | --- | --- | --- | --- | --- | --- |
| *NIPA2* | rs3812922 | 15:22866361 | A | C | 0.0330 | 1.004 | 0.0941 | 0.9687 |
| *SNRPN* | rs8037745 | 15:24902158 | A | G | 0.5963 | 1.016 | 0.0291 | 0.5909 |
| *ATP10A* | rs4906771 | 15:25787248 | T | C | 0.4736 | 0.980 | 0.0280 | 0.4665 |
|  | rs1345098 | 15:25812475 | T | G | 0.5488 | 1.043 | 0.0275 | 0.1251 |
| *GABRB3* | rs12438141 | 15:26625455 | T | C | 0.1372 | 1.025 | 0.0388 | 0.5285 |
|  | rs1863467 | 15:26688587 | T | C | 0.5831 | 0.970 | 0.0275 | 0.2685 |
|  | rs4906902 | 15:26774621 | A | G | 0.8061 | 1.000 | 0.0364 | 0.9993 |
| *GABRG3* | rs7180500 | 15:27008032 | A | C | 0.4947 | 0.938 | 0.0279 | **0.0205** |

Abbreviations: Ref, reference allele for OR; Alt, alternate allele; Freq, frequencies of reference alleles in European descent; OR, odds ratio; SE, standard error.

^a^ *p* value below 0.05 was noted with bold character.
